# Isolation and characterization of SSR and EST‐SSR loci in *Chamaecyparis formosensis* (Cupressaceae)

**DOI:** 10.1002/aps3.1175

**Published:** 2018-08-27

**Authors:** Chiun‐Jr Huang, Fang‐Hua Chu, Shau‐Chian Liu, Yu‐Hsin Tseng, Yi‐Shiang Huang, Li‐Ting Ma, Chieh‐Ting Wang, Ya Ting You, Shuo‐Yu Hsu, Hsiang‐Chih Hsieh, Chi‐Tsong Chen, Chi‐Hsiang Chao

**Affiliations:** ^1^ School of Forestry and Resource Conservation National Taiwan University No. 1, Sec. 4, Roosevelt Road Taipei 10617 Taiwan; ^2^ Department of Forensic Science Investigation Bureau Ministry of Justice No. 74, Zhonghua Road New Taipei City 23149 Taiwan; ^3^ Research Museum and Herbarium (HAST) Biodiversity Research Center Academia Sinica No. 128, Sec. 2, Academia Road Taipei 11529 Taiwan; ^4^ Experimental Forest College of Bioresources and Agriculture National Taiwan University No. 12, Sec. 1, Qianshan Road, Nantou County Taiwan 55750 Taiwan; ^5^ Department of Applied Science National Taitung University No. 369, Sec. 2, University Road Taitung 95092 Taiwan; ^6^ Institute of Biological Chemistry Academia Sinica No. 128, Sec. 2, Academia Road Taipei 11529 Taiwan

**Keywords:** *Chamaecyparis formosensis*, Cupressaceae, expressed sequence tag–simple sequence repeat (EST‐SSR) marker, simple sequence repeat (SSR) marker

## Abstract

**Premise of the Study:**

Simple sequence repeat (SSR) and expressed sequence tag (EST)–SSR markers were developed as tools for marker‐assisted selection of *Chamaecyparis formosensis* and for the molecular differentiation of cypress species.

**Methods and Results:**

Based on the SSR‐enriched genomic libraries and transcriptome data of *C. formosensis*, 300 primer pairs were selected for initial confirmation, of which 19 polymorphic SSR and eight polymorphic EST‐SSR loci were chosen after testing in 92 individuals. The number of alleles observed for these 27 loci ranged from one to 17. The levels of observed and expected heterozygosity ranged from 0.000 to 1.000 and from 0.000 to 0.903, respectively. Most markers also amplified in *C. obtusa* var. *formosana*.

**Conclusions:**

The developed SSR and EST‐SSR sequences are the first reported markers specific to *C. formosensis*. These markers will be useful for individual identification of *C. formosensis* and to distinguish cypress species such as *C. obtusa* var. *formosana*.

*Chamaecyparis formosensis* Matsum., known as cypress, is a coniferous plant species in the Cupressaceae that is endemic to Taiwan. Although several simple sequence repeat (SSR) markers of *Chamaecyparis* Spach have been reported (Nakao et al., 2001; Matsumoto et al., 2006), these markers were not applicable to *C. formosensis* as evidenced in our preliminary screening tests. In the present study, next‐generation sequencing was used to develop two types of effective markers in *C. formosensis*: (1) SSR markers (codominant markers that are theoretically distributed throughout the genome) were developed from noncoding regions, and (2) expressed sequence tag (EST)–SSR markers (which are thought to be highly conserved in closely related species) were derived from functional sequences. Compared to SSR markers, EST‐SSR markers demonstrate a higher level of transferability across related species (Varshney et al., 2005). Thus, EST‐SSR markers are more suitable for the discrimination of species.

The logging of illegally sourced timber poses a great threat to biodiversity. To address this problem, scientists and forestry experts have been developing methods to identify individual trees (Dormontt et al., 2015). Tereba et al. (2017) reported SSR‐based markers to identify and match logs to the stumps at a given locality. Lowe et al. (2010) also demonstrated that SSR markers allow log suppliers to validate the integrity of wood products within a supply chain. The markers developed in this study will be used not only for the individual identification of *C. formosensis*, but also to supply an identification tool for evidence of illegal logging. In addition, we also tested the transferability of these markers in *C. obtusa* (Siebold & Zucc.) Endl. var. *formosana* (Hayata) Hayata to effectively distinguish *C. formosensis* and *C. obtusa* var. *formosana*, which are currently difficult to differentiate by phenotype.

## METHODS AND RESULTS

Marker development was based on a combination of RNA and DNA libraries. To create three DNA libraries, genomic DNA was extracted from fresh leaves using the cetyltrimethylammonium bromide (CTAB) method (Doyle and Doyle, 1987) from three individuals (*Chung 2434*,* Chung 2607*, and *Chung 2626*) from two localities in Taiwan (Appendix 1). Development of the SSR markers from the DNA library followed the magnetic bead enrichment method of Glenn and Schable (2005), using the restriction enzymes *Alu*I, *Xmn*I, and *Hae*III (New England Biolabs, Ipswich, Massachusetts, USA). The concentration and quality of SSR‐enriched libraries were measured using a NanoDrop 2000 (Thermo Fisher Scientific, San Diego, California, USA) and Qubit 2.0 Fluorometer (Thermo Fisher Scientific), respectively, and then the DNA libraries were sequenced using the Illumina MiSeq System (2 × 300 bp paired‐end; Illumina, San Diego, California, USA) at Tri‐I Biotech (New Taipei City, Taiwan). A total of 13,653,074 raw reads were produced. The raw reads were quality‐trimmed and merged using CLC Genomics Workbench version 7.5 (QIAGEN, Aarhus, Denmark). All sequence information has been uploaded to the National Center for Biotechnology Information (NCBI) Sequence Read Archive (SRP145153). The contigs ranging from 80 to 530 bp in length were merged, and a total of 10,487,858 contigs were assembled. These contigs were screened using the Simple Sequence Repeat Identification Tool (SSRIT; Temnykh et al., 2001) and at least five di‐, tri‐, tetra‐, penta‐, and hexanucleotide repeats were selected, resulting in a total of 305,556 SSR‐containing sequences.

To prepare the RNA library, RNA was extracted from fresh leaves of one individual (specimen *C.T. Wang s.n*.) using the CTAB method (Chang et al., 1993). The concentration and quality of total RNA were measured using the NanoDrop 2000 and Qubit 2.0 Fluorometer, respectively, and sequencing was performed via the Illumina HiSeq 2000 System (2 × 100 bp paired‐end) by BGI Genomics (Shenzhen City, Guangdong Province, China). The adapter contamination and low‐quality reads were removed by BGI Genomics. All sequence information has been deposited in the NCBI Sequence Read Archive (SRP145033). There were a total of 48,126,630 clean reads with 90 bp per read. Clean reads were assembled and merged into a single sequence 1,197,968 bp in length using Geneious version 10.2.3 (Biomatters Ltd., Auckland, New Zealand). pSTR Finder (Lee et al., 2015) was used to screen the EST‐SSR sequences, and at least five di‐, tri‐, tetra‐, penta‐, and hexanucleotide repeats were subsequently selected to generate a total of 112 potential EST‐SSR sequences.

Primers were designed for the potential SSR and EST‐SSR sequences using Primer3 (Rozen and Skaletsky, 1999) with the optimum primer conditions: length of 18 to 28 bp, annealing temperature of 45–60°C, and target product size of 80–300 bp. Consequently, a total of 274 SSR primer pairs and 26 EST‐SSR primer pairs were designed. To characterize the degree of polymorphism of each locus, 92 individuals from four populations (Appendix 1) were tested using the primer pairs. For this purpose, total genomic DNA was extracted from frozen leaves or wood samples using the Plant Genomic DNA Extraction Miniprep System Kit (Viogene, Taipei, Taiwan). PCR was conducted with a final volume of 20 μL containing approximately 2 ng of genomic DNA, 0.3 μL each of forward and reverse primer (10 μM), and 10 μL of Q‐Amp 2× Screening Fire Taq Master Mix (Bio‐Genesis Technologies, Taipei, Taiwan). The following PCR conditions were used: an initial denaturation of 95°C for 2 min; 30 cycles of 95°C for 45 s, a primer‐specific annealing temperature (see Tables 1, 2) for 45 s, and 72°C for 45 s; followed by a 15‐min extension at 72°C (Table 1). The amplified products were evaluated on the ABI 3130xl (Applied Biosystems, Waltham, Massachusetts, USA) with GeneScan 500 ROX Size Standard (Applied Biosystems). Fragment sizes were determined by using GeneMapper version 3.2 (Applied Biosystems).

**Table 1:** Characteristics of 19 SSR loci developed in *Chamaecyparis formosensis*

Locus	Primer sequences (5′–3′)	Repeat motif	Fluorescent label	Allele size (bp)	*T*_a_ (°C)	GenBank accession no.
Cred35	F: GGAGAAAGGAGTGTCACAAG	(GATA)_10_	FAM	193	TD58–55	MG807617
	R: AACTCATTCCTTCTCCCTCT					
Cred47	F: CCTCTCTCTCACCCCTCTAT	(TATC)_8_	JOE	153	TD58–55	MG807618
	R: TCTGTATGAGTGTTGCTCCA					
Cred88	F: GCTTCATCGTCCCTAAGTT	(TATC)_5_	FAM	130	TD58–55	MG807619
	R: TTCTGTTCTTGCAAATTGTT					
Cred211	F: AAAAATATCAAGCAGATTACCTCTA	(AAG)_8_	FAM	110	49	MG807620
	R: TCTTTCTTATCTTTTTCTTT					
Cred220	F: CACTGATCTTATGGAGACCATACT	(GAT)_12_	FAM	124	49	MG807621
	R: ATCCATCCCTACAATCCTTAC					
Cred224	F: CACTGACAAACTATCTCCACAG	(AG)_14_	FAM	100	57	MG807622
	R: TAATATCCAGTGTTGTTGACC					
Cred225	F: GGGTTTCTCTCCTACCATTT	(AG)_18_	FAM	101	57	MG807623
	R: TGAGATGCATTAGATTAGAGG					
Cred226	F: CTAGCTCTTCTTCTGGTTGC	(TTC)_22_	FAM	169	57	MG807624
	R: AAAGATATTGATAAAGCAGAAACA					
Cred229	F: GGAGAAAGGAGTGTCACAAG	(GATA)_10_	FAM	130	49	MG807625
	R: CTCTATTTATATCCCATTTCCTCT					
Cred231	F: TACTCAGAAGTGACAACACAAA	(GA)_20_	FAM	111	57	MG807626
	R: GGCATGTATGAATCTTGTGA					
Cred236	F: GGGCATACACTCCACTTAAA	(CA)_22_	FAM	119	57	MG807627
	R: GGATTTGTGTTTCCATAAGGT					
Cred242	F: GAGGGAAAGACAGATGGATA	(TAGA)_11_	FAM	110	54	MG807628
	R: TCTTCTAATATCACTTCCACTC					
Cred248	F: TCCATTTCCTAGACTTACCG	(ATG)_8_	FAM	102	57	MG807629
	R: GCACTACCCATCAGTTATCAA					
Cred249	F: AGCACACTTAATAATAGGATAGA	(AG)_18_	FAM	104	57	MG807630
	R: TGATTTCAATGAGGTATTTCC					
Cred250	F: GGCAAGGTATGACTTTCATT	(ACA)_9_	FAM	108	45	MG807631
	R: TGATTCATGATATTGTTTGTACC					
Cred253	F: TTTCCTCAGATCTTGCTTA	(CTT)_14_	FAM	110	49	MG807632
	R: AAGGAAAGAGGAAACCTGAA					
Cred260	F: CCTTTCAAACATACACTCAAA	(GA)_14_	FAM	116	45	MG807633
	R: GCCCAACATGTATGAAGTTT					
Cred262	F: GACCTTATTGATGTGGATGAA	(TC)_26_	FAM	142	56	MG807634
	R: CAAACAAGATGATATGTGTATTAAA					
Cred264	F: TTCTATAATAGGGGCAGCTT	(TGA)_15_	FAM	124	56	MG807635
	R: CCTTCTAAAAGTAGAACCCAAG					

Note

*T*_a_ = annealing temperature; TD = touchdown PCR.

**Table 2:** Characteristics of eight EST‐SSR loci developed in *Chamaecyparis formosensis*

Locus	Primer sequences (5′–3′)	Repeat motif	Fluorescent label	Allele size (bp)	*T*_a_ (°C)	GenBank accession no.	Putative function [organism]
Cred276	F: CCTTCCTAAGCGGTTCGTG	(AACAGG)_4_	FAM	112	56	MG807636	No hit
	R: CCATCCATCCCTTTCTTTCA						
Cred277	F: CCCTTCTTCGCCATCTTCTT	(CTTCTC)_3_	FAM	165	62	MG807637	No hit
	R: CAGAAAGACAAACCGAAAGACA						
Cred280	F: GCAATGTCTTGAAGCGCTTATC	(TTG)_19_	FAM	150	56	MG807638	No hit
	R: ATACCAATTCAACAATTCATCACAA						
Cred281	F: GTGGAGGAGAAGGTGAAGGA	(ATGGGC)_4_	FAM	140	56	MG807639	No hit
	R: CCCTGAGATTGCCACAGTAG						
Cred295	F: CTCCTCCATGGCCGTGTC	(ATGCCC)_6_	FAM	115	56	MG807640	No hit
	R: CCCTTACCTCGTGAGAAAGATT						
Cred297	F: GTGGAGGAGAAGGTGAAGGA	(ATGGGC)_4_	FAM	116	56	MG807641	No hit
	R: TAACTGCAGAGGCAGAGCAG						
Cred298	F: CTCAGGCGCATACTGTACCA	(TA)_32_	FAM	173	56	MG807642	No hit
	R: CTAATGCAGGTGCAGAAACAG						
Cred299	F: GCCATAGCTACCACCACCAC	(CCG)_8_	FAM	100	62	MG807643	No hit
	R: AGATGTCACTGGCCCTAATGG						

Note

*T*_a_ = annealing temperature.

Of the 274 SSR and 26 EST‐SSR primer pairs, 19 SSR loci and eight EST‐SSR loci were developed (Tables 1, 2) and confirmed to be polymorphic among the four tested populations (Table 3). All sequence information was combined and deposited at NCBI (BioProject PRJNA454510). The number of alleles per locus and levels of expected and observed heterozygosity were calculated using GenAlEx 6.503 (Peakall and Smouse, 2012). GENEPOP 4.2 (Raymond and Rousset, 1995) was used to test for Hardy–Weinberg equilibrium and linkage disequilibrium using exact tests. The total number of alleles ranged from one to 17 (Table 3). The levels of observed and expected heterozygosity ranged from 0.000 to 1.000 and from 0.000 to 0.903, with average values of 0.549 and 0.568, respectively. Significant deviations of Hardy–Weinberg equilibrium in terms of heterozygosity deficiency were detected in 11 loci (Cred47, Cred231, Cred248, Cred249, Cred253, Cred260, Cred262, Cred264, Cred276, Cred277, and Cred280). Significant linkage disequilibrium (*P* < 0.001) was detected between Cred35 and Cred229, Cred281 and Cred297, Cred249 and Cred298, and Cred260 and Cred298. The putative functions of EST‐SSR sequences were determined by BLASTX against the non‐redundant GenBank database. Thirteen SSR and four EST‐SSR loci were successfully amplified in *C. obtusa* var. *formosana* (Table 4).

**Table 3:** Genetic characterization of 27 newly developed polymorphic SSR and EST‐SSR loci of *Chamaecyparis formosensis*.^a^

Locus	MM (*N* = 20)			HV (*N* = 25)			GW (*N* = 23)			SY (*N* = 24)		
	*A*	*H*_o_	*H*_e_	*A*	*H*_o_	*H*_e_	*A*	*H*_o_	*H*_e_	*A*	*H*_o_	*H*_e_
Cred35	4	0.500	0.441	5	0.400	0.398	3	0.304	0.373	6	0.792	0.708
Cred47	6	0.200	0.570*	4	0.320	0.442*	3	0.261	0.349	12	0.417	0.871*
Cred88	3	0.500	0.486	2	0.360	0.385	2	0.478	0.466	2	0.458	0.430
Cred211	3	0.800	0.629	4	0.400	0.342	3	0.870	0.655	4	0.667	0.635
Cred220	4	0.300	0.303	4	0.720	0.666	6	0.783	0.616	4	0.625	0.497
Cred224	7	0.750	0.723	6	0.880	0.765	7	0.609	0.707	9	0.667	0.742
Cred225	10	0.750	0.718	9	0.960	0.834	10	0.913	0.863	9	0.792	0.655
Cred226	3	0.500	0.516	5	0.640	0.734	6	0.609	0.579	4	0.667	0.635
Cred229	4	0.450	0.415	6	0.440	0.377	5	0.435	0.474	5	0.708	0.701
Cred231	8	0.750	0.825	5	0.480	0.640	10	0.304	0.840*	10	0.792	0.861
Cred236	15	0.850	0.898	11	0.720	0.829	17	0.870	0.883	10	0.917	0.744
Cred242	5	0.550	0.576	4	0.360	0.620	5	0.217	0.586	5	0.625	0.619
Cred248	6	0.700	0.560	5	0.440	0.450	4	0.435	0.422	7	0.833	0.636*
Cred249	5	0.450	0.693	7	0.320	0.511*	9	0.522	0.820	6	0.250	0.725*
Cred250	3	0.400	0.421	4	0.680	0.618	3	0.130	0.267	6	0.500	0.651
Cred253	9	0.750	0.833	6	0.440	0.679*	10	0.826	0.838	10	0.875	0.832
Cred260	4	0.500	0.464	4	0.680	0.687	8	0.696	0.732	7	0.917	0.670*
Cred262	10	0.700	0.779	12	0.840	0.826*	16	0.870	0.903	10	0.917	0.858
Cred264	8	0.600	0.620	8	0.720	0.734	7	0.565	0.578*	6	0.583	0.681
Cred276	2	1.000	0.500*	2	1.000	0.500*	3	0.696	0.480	3	0.875	0.582
Cred277	5	0.150	0.639*	4	0.120	0.638*	7	0.391	0.488*	7	0.417	0.694
Cred280	1	0.000	0.000	2	0.160	0.147	3	0.217	0.199	4	0.583	0.523*
Cred281	2	0.250	0.289	2	0.120	0.113	2	0.087	0.083	3	0.833	0.573
Cred295	3	0.350	0.484	2	0.520	0.497	3	0.522	0.499	4	0.542	0.619
Cred297	1	0.000	0.000	1	0.000	0.000	1	0.000	0.000	2	0.417	0.330
Cred298	6	0.750	0.731	6	0.880	0.703	14	0.870	0.843	9	0.917	0.836
Cred299	1	0.000	0.000	3	0.400	0.447	2	0.174	0.287	4	0.583	0.513

Note

*A* = number of alleles; *H*_e_ = expected heterozygosity; *H*_o_ = observed heterozygosity; *N* = number of individuals sampled.

*Highly significant deviation from Hardy–Weinberg equilibrium (*P* < 0.001).

^a^Locality and voucher information for the populations are provided in Appendix 1.

**Table 4:** Cross‐amplification results for the 19 SSR and eight EST‐SSR loci developed in *Chamaecyparis formosensis* in eight populations of *C. obtusa* var. *formosana*.^a^

Locus	TP	CF	NC	DS	GW	LL	QL	FR
Cred35	+	+	+	+	+	+	+	+
Cred47	—	—	—	—	—	—	—	—
Cred88	—	—	—	—	—	—	—	—
Cred211	+	+	+	+	+	+	+	+
Cred220	+	+	+	+	+	+	+	+
Cred224	—	+	+	—	+	+	+	+
Cred225	+	+	+	+	+	+	+	+
Cred226	+	+	+	+	+	+	+	+
Cred229	+	+	+	+	+	+	+	+
Cred231	+	+	+	+	+	+	+	+
Cred236	+	+	+	+	+	+	+	+
Cred242	—	—	—	—	—	—	—	—
Cred248	+	+	+	+	+	+	+	+
Cred249	+	+	+	+	+	+	+	+
Cred250	—	+	+	+	+	—	—	+
Cred253	+	+	+	+	+	+	+	+
Cred260	+	+	+	+	+	+	+	+
Cred262	—	+	—	—	+	—	—	—
Cred264	+	+	+	+	+	+	+	+
Cred276	+	+	+	+	+	+	+	+
Cred277	+	+	+	+	+	+	+	+
Cred280	+	+	+	+	+	+	+	+
Cred281	—	+	+	—	+	+	+	+
Cred295	+	—	—	—	—	—	—	—
Cred297	—	+	—	—	—	+	+	—
Cred298	—	+	+	+	+	+	+	+
Cred299	+	+	+	+	+	+	+	+

Note

+ = successful amplification; — = failed amplification.

a Locality and voucher information for the populations are provided in Appendix 1.

## CONCLUSIONS

The 19 SSR and eight EST‐SSR markers described in the present study are reported for the first time in *C. formosensis*. These endemic cypress–specific markers can be used not only for species identification, but also potentially to assist in the certification of legal timber trade and in studies of genetic diversity and population genetic structure in populations within Taiwan. Data from these types of studies will contribute to the conservation and management of *C. formosensis*, which is crucially threatened by illegal logging.

## DATA ACCESSIBILITY

Raw sequence information has been deposited to the National Center for Biotechnology Information (NCBI) Sequence Read Archive (DNA sequence information: SRP145153; RNA sequence information SRP145033). Sequence information for the developed SSR and EST‐SSR primer pairs has been deposited to NCBI (BioProject ID PRJNA454510); GenBank accession numbers are provided in Tables 1 and 2.

## APPENDIX 1 Voucher information for *Chamaecyparis formosensis* and *C. obtusa* var. *formosana* individuals used in this study. All voucher specimens are deposited in the Herbarium of the Biodiversity Research Center (HAST), Academia Sinica, Taipei, Taiwan.

**Table:**

Species	Voucher no.	Collection locality	Geographic coordinates	Population code	*N*
*Chamaecyparis formosensis* Matsum.	*Chung 2434*	Taipingshan Forest Recreation Area, Datong Township, Yilan County 267, Taiwan (R.O.C.)	24°29′40.96″N, 121°32′6.59″E	TP	1
	*Chung 2607*,* 2626*	No. 100 Forest Rd., Jianshi Township, Hsinchu County 313, Taiwan (R.O.C.)	24°35′30.00″N, 121°25′12.00″E	—	2
	*C.T. Wang s.n*.	XITOU Nature Education Area, Lugu Township, Nantou 558, Taiwan (R.O.C.)	23°39′38.12″N, 120°47′54.41″E	—	1
	*Chung 3143, 3144, 3145, 3158, 3161, 3165, 3167, 3168, 3169, 3170, 3171, 3172, 3174, 3177, 3178, 3180, 3181, 3182, 3183, 3184*	Meli‐miligang, Taiwu Township, Pingtung County 921, Taiwan (R.O.C.)	22°36′53.37″N, 120°44′26.05″E	MM	20
	*Chung 3185, 3186, 3187, 3188, 3189, 3192, 3193, 3194, 3195, 3196, 3198, 3201, 3202, 3203, 3204, 3205, 3206, 3210, 3211, 3212, 3214, 3215, 3216, 3217, 3218*	Herve Divine Trees, Fuxing Dist, Taoyuan City 336, Taiwan (R.O.C.)	24°47′27.13″N, 121°26′14.86″E	HV	25
	*Chung 4007, 4008, 4009, 4010, 4011, 4012, 4013, 4015, 4016, 4017, 4018, 4022, 4023, 4025, 4026, 4027, 4028, 4030, 4031, 4032, 4033, 4034, 4035*	Guanwu Forest Recreation Area, Tai'an Township, Miaoli County 365, Taiwan (R.O.C.)	24°30′6.18″N, 121°05′30.66″E	GW	23
	*Chung 4254, 4255, 4256, 4257, 4258, 4259, 4260, 4261, 4262, 4263, 4264, 4265, 4266, 4268, 4269, 4277, 4281, 4282, 4284, 4285, 4286, 4287, 4288, 4289*	Siangyang Forest Recreation Area, Haiduan Township, Taitung Country 957, Taiwan (R.O.C.)	23°15′1.40″N, 120°59′8.54″E	SY	24
*C. obtusa* (Siebold & Zucc.) Endl. var. *formosana* (Hayata) Hayata	*Chung 2435*	Taipingshan Forest Recreation Area, Datong Township, Yilan County 267, Taiwan (R.O.C.)	24°29′40.96″N, 121°32′6.59″E	TP	1
	*Chung 2476*	Cueifong Lake, Datong Township, Yilan County 267, Taiwan (R.O.C.)	24°30′37.45″N, 121°36′32.52″E	CF	1
	*Chung 3116*	No. 7 provincial hwy., Yilan City, Yilan County 260, Taiwan (R.O.C.)	24°38′40.08″N, 121°26′39.47″E	NC	1
	*Chung 3241*	Dasyueshan Forest Recreation Area, Heping Dist., Taichung City 424, Taiwan (R.O.C.)	24°13′9.59″N, 120°53′9.06″E	DS	1
	*Chung 4021*	Guanwu Forest Recreation Area, Tai'an Township, Miaoli County 365, Taiwan (R.O.C.)	24°30′6.18″N, 121°05′30.66″E	GW	1
	*Chung 4190*	Lalashan Forest Recreation Area, Fuxing Dist, Taoyuan City 336, Taiwan (R.O.C.)	24°32′17.04″N, 121°17′40.03″E	LL	1
	*Chung 4432*	Qilan Forest Recration Area, Datong Township, Yilan Country 267, Taiwan (R.O.C.)	24°35′26.28″N, 121°26′15.34″E	QL	1
	*Chung 4541*	No. 160 Forest Rd., Jianshi Township, Hsinchu County 313, Taiwan (R.O.C.)	24°32′22.04″N, 121°22′40.74″E	FR	1

Note

*N* = number of individuals sampled.
